# Preclinical and Clinical Therapeutic Strategies Affecting Tumor-Associated Macrophages in Hepatocellular Carcinoma

**DOI:** 10.1155/2018/7819520

**Published:** 2018-10-16

**Authors:** H. Degroote, A. Van Dierendonck, A. Geerts, H. Van Vlierberghe, L. Devisscher

**Affiliations:** ^1^Department of Gastroenterology and Hepatology, Ghent University, Belgium; ^2^Ghent University Hospital, Building K12 First Floor IE, Corneel Heymanslaan 10, 9000 Ghent, Belgium; ^3^Department Basic and Applied Medical Sciences, Ghent University, Belgium

## Abstract

Hepatocellular carcinoma (HCC) most often develops in patients with underlying liver disease characterized by chronic nonresolving inflammation. Tumor-associated macrophages (TAMs) are one of the most abundant immune cell populations within the tumoral microenvironment. As key actors of cancer-related inflammation, they promote tumor growth by suppression of effective anticancer immunity, stimulation of angiogenesis, and tissue remodeling. Therefore, they have become an attractive and promising target for immunotherapy. The heterogeneity of TAM subtypes and their origin and dynamic phenotype during the initiation and progression of HCC has been partially unraveled and forms the base for the development of therapeutic agents. Current approaches are aimed at decreasing the population of TAMs by depleting macrophages present in the tumor, blocking the recruitment of bone marrow-derived monocytes, and/or functionally reprogramming TAMs to antitumoral behavior. In this review, the preclinical evolution and hitherto clinical trials for TAM-targeted therapy in HCC will be highlighted.

## 1. Introduction

Hepatocellular carcinoma (HCC) lesions usually arise in patients with underlying liver cirrhosis, characterized by a chronic, dysregulated inflammatory environment that predisposes to cancer initiation. In chronic liver diseases, a predominantly proinflammatory state switches to persistent systemic inflammation and immune cell stimulation but with impairment of specific immune responses such as phagocytosis and antigen-presenting ability, a condition which is called cirrhosis-associated immune dysfunction. This is an important difference with an acute inflammatory response, regarded as protective and beneficial in the acute setting of liver damage and resolution. Chronic inflammation drives indeed a maladaptive tissue repair reaction and eventually results in the development of dysplastic nodules and cancer [1–4].

The central functions of macrophages during chronic liver diseases include the perpetuation of chronic inflammation and hepatocyte injury, activation of hepatic stellate cells with subsequent fibrogenesis, and support of tumor development by providing cytokines, chemokines, growth factors, and matrix metalloproteases, all of which are factors that favor angiogenesis and tumor cell proliferation and protection from cancer cell apoptosis and metastasis [5, 6]. Thus, hepatic macrophages provide a tumor-prone inflammatory microenvironment and at the same time respond to tumor and other stromal cell-derived signals to actively facilitate HCC progression [1, 7, 8]. Tumor-associated macrophages (TAMs) also stimulate tumor growth by acting as immune suppressor cells of the adaptive system. Not only do TAMs exhibit generally low antigen-presenting and costimulating capacity but they also actively support cancer cells to evade antitumor immunity by secreting anti-inflammatory cytokines and activating T cell checkpoint blockade. In this regard, tumor-infiltrating monocytes in HCC express high level of programmed cell death-ligand 1 (PD-L1) that binds with PD-1 on CD8+ T cells and suppresses antitumoral cytotoxic T cell responses [9–11]. This overall immune-suppressive effect is reinforced by cross-talk with other important immune cells in the tumoral microenvironment, such as myeloid-derived suppressor cells (MDSC) and regulatory T cells (Tregs). Besides t5he suppression of cytotoxic T cells, MDSC and Tregs contribute to the dysfunctional state of dendritic cells (DCs) [1, 12–14].

Since TAMs influence various aspects of cancer progression, novel strategies to treat HCC are aimed at targeting tumor-promoting macrophages. New therapeutic development is an urgent unmet need as options are still limited for patients with advanced HCC or earlier stage progressing upon or patients unsuitable for locoregional therapies. Nowadays, cancer immunotherapy mainly focuses on immune checkpoint inhibitors. After the observed efficacy in other solid tumors, clinical trials are currently ongoing to evaluate the utility in patients with HCC. Based on promising data in the phase I/II CheckMate-040 trial, immune therapy with nivolumab (anti-PD-1 antibody) has received FDA approval in second-line treatment [15]. However, only 20% of patients are responsive. In solid tumors, recent studies suggest that the efficacy could be enhanced using coordinated strategies to counteract the TAM-dependent impairment of immune adaptive responses [16–18].

Although the clinical application of a TAM-targeted approach still has to be determined, a number of experimental preclinical studies have shown promising effects. Most studies involve other solid tumors and are described elsewhere [19–24]. In this review, the preclinical progress and limited clinical trials affecting TAMs in HCC therapy will be highlighted. Furthermore, the encountered challenges are discussed in relation to fundamental insights into the heterogeneity of TAM subtypes and their origin and dynamic phenotype and function during the initiation and progression of HCC. Lastly, we elaborate on the potential contributive effect of combinational therapies with clinically used therapies such as sorafenib and immune checkpoint inhibitors.

## 2. Definition and Origin of TAMs in HCC

Liver macrophages consist of ontogenically distinct populations, namely, the resident Kupffer cells (KCs) and monocyte-derived macrophages (Mo-Mfs). Kupffer cells are self-renewing and nonmigratory phagocytes. They originate from yolk sac-derived specific progenitor cells that seed the liver during embryogenesis. In the tumoral microenvironment, chemokines secreted by malignant and stromal cells recruit bone marrow-derived Ly-6c^hi^ monocytes. These infiltrating monocytes subsequently give rise to large numbers of Mo-Mfs. Monocyte-derived macrophages further differentiate and can replace and acquire a phenotype that is almost indistinguishable from resident KCs under specific circumstances [25–30]. After infiltration, Mo-Mfs even seem to acquire the ability to proliferate [27]. It is however unclear if they are able to sustain the number of TAMs in tumor lesions independently from recruitment. As a result of this continuous transition, the compartment of hepatic myeloid cells consists of subtypes of macrophages in a different state of differentiation. Each state is associated with stereotypic alterations in cell surface marker expression, which can be used for identification. In many studies, CD68 is used as an indicator for tissue macrophages, but this marker is not sufficiently specific. More recently, two markers were proposed to distinguish between Mo-Mfs and KCs. Clec4F and Tim4 are expressed by KCs but absent from infiltrating Mo-Mfs. Additionally, these markers can be used to discriminate between KCs and recently differentiated Mo-KCs as the latter do not express Tim4 in the first week postdifferentiation. However, with time, Mo-KCs will also gain expression of Tim4 [27, 28].

It is not clear to what extent TAMs are derived from tissue-resident liver cells or only represent infiltrating bone-marrow derived Mo-Mfs. In most reports, macrophages present in the tumoral microenvironment are considered and classified as “tumor-associated macrophages.” Although KCs were initially thought to be only involved in antitumor immunity, there is substantial evidence that suggests that KCs are part of the TAM population and enhance tumor progression [3, 31–33]. KCs are triggered by damage-associated molecular patterns (DAMPs) released from damaged liver cells and pathogen-associated molecular patterns (PAMPs), mostly derived from the gut due to alterations in gut microbiota composition and/or increased intestinal permeability. The liver is supplied with blood via the portal vein from the intestinal tract and via hepatic arteries from the blood circulation. As such, KCs in the liver sinusoids are exposed to bacteria and associated toxins from the bloodstream [34]. DAMPs and PAMPs interact with pattern recognition receptors (PRR) on KC or directly on activating inflammasomes [34]. For example, the interaction of lipopolysaccharide (LPS) with Toll-like receptor 4 (TLR-4) on KCs showed stimulation of cancer-promoting signaling pathways in mice [35].

## 3. Phenotype and Function of TAMs in HCC

Defining TAMs as one population has limitations as shown in the contradictory results of prognostic studies, summarized in Table 1. This is mostly due to an overgeneralized definition of TAMs and indicates the need for further subdivision according to their polarization. Polarization refers to how macrophages have been activated as they can rapidly adapt to their phenotype according to signals derived from the hepatic microenvironment. Macrophages have been assigned a classically activated (proinflammatory) M1 state triggered by interferon-*γ* and/or lipopolysaccharide or an alternatively activated (anti-inflammatory) M2 state induced by IL-4. This traditional nomenclature however is derived from in vitro studies and does not represent chronic inflammation or the complex tumoral microenvironment. Moreover, the expression and secretory profile of macrophage subsets are not dichotomous and can differ according to the model and method of inducing polarization. There is also considerable difference between mouse and human cells in terms of molecules associated with macrophage polarization [28, 36–39].

The pro- and anti-inflammatory paradigm leads to the confusing assumption that in an inflammation-related tumor, an M2 phenotype would be beneficial. However, during tumor progression in HCC, macrophage function is skewed from M1 to M2 phenotype [10, 40]. An anti-inflammatory phenotype does not result in the resolution of inflammation but refers to the immune-deficient and immune-suppressive state of these macrophages and consequently immune evasion of cancer cells. On the other hand, a proinflammatory phenotype does not refer to inflammatory damage in an acute setting but represents a coordinated immune attack of tumor cells. Thus, the M1/M2 model is too simplistic to describe the polarization of liver macrophages in cancer. Currently, TAMs are most often defined as M1-like (leading to antitumor responses and cytotoxicity) or M2-like (tumor promotion and suppression of effective adaptive immunity) cells, taking into account the relative proportion between both characteristics as they often simultaneously express markers of both ends of the continuum [36]. The polarization of macrophages not only depends on the disease stage but also differs between tumoral nodules or within different areas of the same tumor. In human HCC, for example, most of the macrophages that are localized perivascularly are more M1-like compared to the M2-like TAM in hypoxic areas [25, 38].

## 4. TAM-Targeted Therapy in HCC

**Figure 1:**
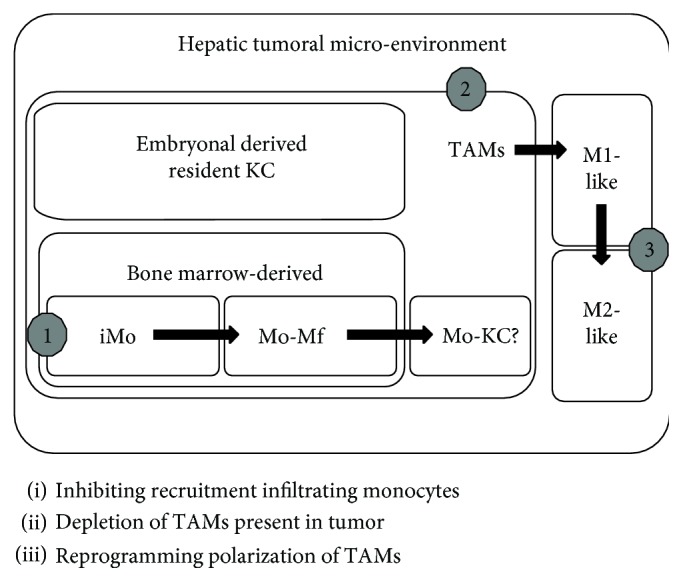
Origin of TAMs in the hepatic tumoral microenvironment and related TAM-targeted strategies.

Current approaches for TAM-targeted therapy are aimed at decreasing the population of TAMs by eliminating TAMs present in the tumor, blocking recruitment of bone marrow-derived monocytes, and/or reprogramming TAM polarization to antitumoral behavior (Figure 1).

### 4.1. Preclinical Studies

In the following section, only preclinical studies in mouse models using agents with a direct effect on TAMs in HCC will be discussed and are summarized in Table 2. Gene therapy or knockout models are beyond the scope of this review.

#### 4.1.1. Depletion of TAMs

Liposomes are artificially prepared vesicles that undergo phagocytosis by macrophages after injection. They can be loaded with clodronate (a bisphosphonate used for osteoporosis) which induces apoptosis of macrophages after intracellular release from the liposomes. Administration of clodronate- (Cl2MDP) encapsulated liposomes partially depleted TAMs (defined as F4/80- and CD68-positive cells on immunohistochemical staining), resulting in reduced tumor growth in a murine Hepa1-6 cell-transplanted tumor model. Not only was the total amount of TAMs reduced but also the number of M2-like TAMs in tumors of liposome-treated mice was significantly lower than that in tumors of untreated mice. In contrast, the number of M1 TAMs was not significantly affected. According to the authors, these results suggest that after depleting the majority of TAMs, the remaining macrophages might undergo a phenotypical transition [52].

Selective depletion of only tumor-promoting macrophages, not just all cells with phagocyting capacity, is an encountered difficulty in TAM-targeted therapy. An excessive reduction of nontumoral macrophages might lead to safety concerns when concomitant infections occur. The use of macrophage subset-specific markers might provide a solution and has successfully been used in the field of imaging where KC-specific [53] or TAM-specific targeting by nanobodies coupled to SPECT or PET tracers allowed KC-specific and M2-like TAM-specific imaging, respectively [54]. Further research is warranted to see if this approach could be translated to pharmaceutical development. It must be emphasized that TAMs are strongly connected with other immune and stromal cells in the microenvironment and it is not clear to what extent other cells will compensate for their function after depletion.

#### 4.1.2. Inhibiting Recruitment of Monocytes

The chemokine C-C motif ligand 2 (CCL2, also referred to as monocyte chemoattractant protein 1 or MCP-1) and the corresponding CCL2-CCR2 signaling axis are important targets to inhibit the recruitment of monocytes. Treatment with a CCR2 antagonist inhibited HCC tumor growth in different murine models. The therapy reduced the infiltration of blood Ly6C^high^ inflammatory monocytes, subsequently lowered the number of TAMs (CD11b- and F4/80-positive cells) in the HCC lesions, and reduced most of the cytokines or chemokines produced by M2-like TAMs (CD206-positive cells). Moreover, the reduced number of remaining TAM shifted towards M1 phenotype. The CCR2 antagonist also supported tumor-infiltrated CD8+ T cells by blocking TAM-mediated immunosuppression [55, 56]. In addition, Teng et al. showed the tumor-inhibiting effect of a CCL2 neutralizing antibody by reducing the population of inflammatory myeloid cells in a HCC mouse model [57]. Although several chemokines are involved in attracting monocytes and targeting one pathway might not completely eliminate recruitment, blocking the CCL2-CCR2 seems to be effective in the inhibition of HCC growth.

Infiltration of monocytes is considered the most important source of TAMs in the tumoral microenvironment. It is still unclear if TAMs are able to sustain their number (or least partially) in tumors by proliferation independently from recruitment or how long TAMs survive in the tumoral microenvironment. Related to this issue, effective timing to start inhibiting recruitment of monocytes can be debated as during early stages, TAMs can also exert an antitumoral function.

#### 4.1.3. Reprogramming Polarization of TAMs

Oral administration of baicalin, a natural flavonoid present in several medicinal plants, inhibited growth of HCC lesions in an orthotopic mouse model by initiating TAM reprogramming to an M1-like phenotype with proinflammatory cytokine production. Coculturing of HCC cells with baicalin-treated macrophages resulted in reduced proliferation and motility in vitro [58].

Colony-stimulating factor-1 (CSF-1) and its receptor, CSF-1R, regulate the differentiation and function of macrophages. CSF-1R blockade by a competitive inhibitor significantly delayed tumor growth in murine xenograft models. The compound inhibited the proliferation of macrophages in vitro, but macrophage infiltration was not decreased in vivo. Thus, the effect is not mediated by TAM depletion. Gene expression profiling showed that TAMs in the treated tumors are polarized towards an M1-like phenotype [59].

An imbalance towards M1-like macrophages might theoretically be harmful by inducing toxicity and inflammatory conditions. In the mentioned studies, no toxic effects were observed but further studies are necessary.

#### 4.1.4. Blocking the Downstream Effect of TAM Products

TAMs represent a major paracrine IL-6 source during HCC progression, and autocrine IL-6 contributed significantly to HCC initiation from HCC progenitor cells. Blockade of IL-6 signaling using tocilizumab, an anti-IL-6 receptor antibody approved by the FDA for the treatment of rheumatoid arthritis, was able to inhibit TAM-stimulated activity of cancer stem cells in vitro and in vivo [60].

### 4.2. Preclinical Therapy Affecting TAMs with Currently Used Clinical Therapies

Sorafenib, an antiangiogenic oral multikinase inhibitor, is currently the standard first-line systemic treatment approved by the US Food and Drug Administration (FDA) and European Medicines Agency (EMA) for patients with advanced HCC. Preclinical studies show that sorafenib interferes with the polarization of TAMs and their cytokine production. In a HepG2 HCC cell line, sorafenib inhibited polarized macrophage-induced epithelial-mesenchymal transition and migration of HCC cells [61]. Administration of sorafenib reduced M2-like TAMs, inhibited their immune-suppressive effect, and stimulated antitumor natural killer (NK) cell responses in both HCC models [62]. In addition, Sprinzl et al. [63] demonstrated a decrease in CD163 serum concentration in 21 patients with HCC during treatment with sorafenib. This finding suggests that Sorafenib suppressed M2 activation in HCC patients, since soluble sCD163 is shedded into serum by activated macrophages and can serve as an indicator to follow M2 macrophage responses [51, 64]. Together, these findings indicate that macrophage modulation contributes to the anticancer activity of sorafenib.

Interestingly, the combination of sorafenib with TAM-targeting agents such as clodronate-loaded liposomes and zoledronic acid (another bisphosphonate used for the treatment of bone metastasis) augmented the inhibitory effect of sorafenib on tumor angiogenesis, growth, and metastasis in HCC xenograft mouse models [65]. A phase II study of sorafenib combined with zoledronic acid in advanced HCC has been conducted (NCT01259193), but no results have been published yet. Besides depletion of macrophages, nitrogen-containing bisphosphonates (such as zoledronic acid) activate *γδ* T cells, potentiating their antitumor function. This immunomodulatory effect of zoledronic acid on *γδ* T cells is exerted through direct or indirect interaction induced by TAMs that endocytose bisphosphonate-encapsulated liposomes [66, 67]. Not only is this effect shown for sorafenib but also the combination of locoregional therapy such as transarterial chemoembolization (TACE) and zoledronic acid treatment showed enhanced therapeutic efficacy with inhibition of TAM infiltration (F4/80+) and tumor angiogenesis in a rat HCC model [68]. The enhanced efficacy of sorafenib together with a CCR2 antagonist to inhibit monocyte infiltration has been shown in a murine HCC model [56].

The effect of checkpoint blockade immunotherapy on TAMs has been shown for other solid tumors, but no data are available in HCC. Blockade of PD-1 in vivo reduced tumor growth and extended the survival in a colorectal cancer mouse model by polarization of TAMs to a phagocytic phenotype [69]. The efficacy of anticytotoxic T lymphocyte-associated antigen 4 (CTLA-4) monoclonal antibodies in a melanoma mouse model and humans is codefined by elimination of regulatory T cells by TAM targeting via antibody-dependent cellular cytotoxicity [16–18, 45]. Also, in a pancreatic cancer mouse model, the combination of depleting M2-like TAMs and repolarization towards antitumoral behavior through blockage of CSF-1/CSF-1R and immunotherapy (PD-1 and CTLA-4 antagonists) reduced tumor progression [18].

### 4.3. Clinical Trials

Glypican-3 is a proteoglycan that is attached to the cell surface and plays an important role in cellular growth, differentiation, and migration. Glypican-3 is highly expressed in HCC tissue and correlates with poor prognosis. It is considered a tumor-derived carcinoembryonic antigen. For example, expression of glypican-3 was associated with upregulation of CCL5, CCL3, and CSF-1 in a HCC xenograft model [70], all of which are chemokines that have been shown to enhance the recruitment of TAMs. Glypican-3 antibodies have been tested in small phase I trials for advanced HCC with promising results (in 13 and 20 patients, respectively). The antibody is well tolerated, and preliminary antitumor activity shows a threefold prolongation of the median time to progression in treated patients with advanced HCC (Child–Pugh A or B cirrhosis) [71, 72]. No phase II trials are currently registered with glypican-3 antibodies for HCC.

## 5. Conclusion

The tumor-promoting cascade of initial injury recognition, amplification of inflammation by monocyte recruitment, and context-dependent differentiation into functionally distinct macrophage populations in the liver offers different approaches for therapeutic interventions in HCC. Although the clinical application of TAM-targeted therapy is still in its infancy, a number of preclinical studies in HCC murine models have shown promising results. The most important obstacles to overcome are firstly the specificity of depleting only protumoral TAMs while not affecting (or even enhancing) antitumor immunity and secondly the perfect balance of their polarization towards antitumoral behavior without toxicity and side effects. The observed potential contributive effect of immune checkpoint inhibitors on solid tumors and currently used clinical therapies for HCC such as sorafenib is encouraging and must be further explored.

## Figures and Tables

**Table 1:** Prognosis of HCC according to TAM identification and polarization. Immunohistochemical staining for CD68, CD86 (M1), or CD163 and CD206 (M2) is frequently used to quantify and classify TAMs [38, 41]. Expression of the used tissue markers was determined by immunohistochemical staining. Serum sCD163 levels were measured by ELISA. Defining TAMs as solely CD68+ cells gives contradictory prognostic results. When however TAMs are subdivided for location (intra- or peritumoral) and polarization (M1- or M2-like cells), a more distinct prognostic value can be attributed. Moreover, it becomes clear from the presented studies that the presence of M2-like oriented TAMs results in a poor prognostic outcome and intratumoral M1-like TAMs correlate with good prognosis.

Author	Number of patient samples	Type of sample	Marker	Definition	Prognosis
Li et al. [42]	101	Intratumoral	CD68+	TAM	Poor
Ding et al. [43]	137	Intratumoral	CD68+	TAM	Poor
Kuang et al. [11]	262	Peritumoral	CD68+	TAM	Poor
Zhang et al. [44]	149	Peritumoral	CD68+	TAM	Poor
Zhou et al. [45]	213	Intratumoral	CD68+	TAM	Poor
Wu et al. [33]	71	Intratumoral	CD68+	TAM	Poor
Minami et al. [46]	105	Intratumoral	CD68+	TAM	Poor
Liao et al. [47]	387	Intratumoral	CD68+	TAM	Not related
Dong et al. [48]	253	Intratumoral	CD68+	TAM	Not related
Yeung et al. [49]	93	Intratumoral	CD68+	TAM	Good
Yeung et al. [49]	93	Peritumoral	CD68+	TAM	Poor
Li et al. [50]	302	Intratumoral	CD68+	TAM	Good
Liao et al. [47]	387	Intratumoral	CD16+	M2-like	Poor
Waidmann et al. [51]	267	Serum	sCD163+	M2-like	Poor
Minami et al. [46]	105	Intratumoral	CD163+	M2-like	Poor
Yeung et al. [49]	93	Peritumoral	CD163+	M2-like	Poor
Dong et al. [48]	253	Intratumoral	CD206+	M2-like	Poor
Dong et al. [48]	253	Intratumoral	CD86+	M1-like	Good

**Table 2:** Preclinical TAM-targeted therapies in HCC.

Author	Product	Mechanism of action	Animal model	Results
Wang et al. [52]	Clodronate-liposomes	Depletion of TAMs	Hepa1-6 HCC cell line xenograft and orthotopic mouse model	Inhibition of tumor growth
Zhang et al. [65]	Sorafenib and zoledronic acid or clodronate-liposomes	Depletion of TAMs	HCCLM3-R and SMMC7721 HCC cell line xenograft mouse models	Inhibition of tumor growth, lung metastasis, and angiogenesis
Li et al. [55]	CCR2 antagonist	Inhibiting recruitment of monocytes M2 polarization of TAMs	Hepa1-6 and LPC-H12 HCC cell line xenograft and Hepa1-6 orthotopic mouse model	Inhibition of tumor growth and metastasis, reduction of recurrence, enhanced survival, and activation of CD8+ T cells
Yao et al. [56]	CCR2 antagonist	Inhibiting recruitment of monocytes	Hepa1-6 or LPC-H12 HCC cell line xenograft and Hepa1-6 orthotopic mouse models	Inhibition of tumor growth, increase in CD8+ T cells, and potentiation effect of sorafenib
Teng et al. [57]	CCR2 monoclonal antibody	Inhibiting recruitment of monocytes	miR-122-knockout HCC mouse model	Inhibition of tumor growth and activation of natural killer cells
Zhou et al. [68]	Sorafenib and TACE	Inhibiting recruitment of monocytes	Walker-256 HCC cell line xenograft and orthotopic rat models	Inhibition of tumor growth and angiogenesis
Tan et al. [58]	Baicalin	Reprogramming polarization of TAMs	MHCC97L HCC cell line orthotopic mouse model	Inhibition of tumor growth
Ao et al. [59]	CSF-1 receptor antagonist	Reprogramming polarization of TAMs	Hepa1-6, HepG2, or HCCLM3 HCC cell line orthotopic mouse model	Delayed tumor growth and increase in CD8+ T cells
Sprinzl et al. [62]	Sorafenib	Reprogramming polarization of TAMs	Hepatitis B virus replicating HBV1.3.32 or albumin-promoter-controlled lymphotoxin-a/b transgenic mice	Activation of natural killer cells and cytotoxicity
Wan et al. [60]	IL-6 receptor monoclonal antibody	Blocking downstream effect of TAM products	HepG2 or human HCC cell line xenograft mouse model	Reduction of tumor growth

## Abbreviations

CSF-1: Colony-stimulating factor-1
CSF-1R: Colony-stimulating factor-1 receptor
CTLA-4: Cytotoxic T lymphocyte-associated antigen 4
DAMP: Damage-associated molecular patterns
HCC: Hepatocellular carcinoma
KCs: Kupffer cells
Mo-Mfs: Monocyte-derived macrophages
Mo-KCs: Monocyte-derived Kupffer cells
PAMPs: Pathogen-associated molecular patterns
PD-1: Programmed cell death 1
PD-L1: Programmed cell death-ligand 1
TAMs: Tumor-associated macrophages.
